# Relationship between electrode position of deep brain stimulation and motor symptoms of Parkinson’s disease

**DOI:** 10.1186/s12883-021-02148-1

**Published:** 2021-03-17

**Authors:** Feng Zhang, Feng Wang, Weiguo Li, Ning Wang, Chunlei Han, Shiying Fan, Peng Li, Lifeng Xu, Jianguo Zhang, Fangang Meng

**Affiliations:** 1 Beijing Neurosurgical Institute, Capital Medical University, Beijing, 100070 China; 2 Department of neurosurgery, the First Hospital of Hebei Medical University, Shijiazhuang, 050031 Hebei China; 3 Departments of Neurosurgery, The First Affiliated Hospital, Zhejiang University School of Medicine, Zhejiang, 310000 Hangzhou China; 4 Department of neurosurgery, QiLu Hospital of Shandong University, Jinan, 250012 China; 5 Department of Neurosurgery, Beijing Tiantan Hospital, Capital Medical University, Beijing, 100070 China; 6 Beijing Key Laboratory of Neurostimulation, Beijing, 100070 China; 7 Chinese Institute for Brain Research, Beijing, 102206 China

**Keywords:** Parkinson’s disease, Deep brain stimulation, Subthalamic nucleus, Motor symptom, Active contacts, Volume of tissue activated

## Abstract

**Background:**

To investigate the relationship between the position of bilateral STN-DBS location of active contacts and the clinical efficacy of STN-DBS on motor symptoms in Parkinson’s disease (PD) patients.

**Methods:**

Retrospectively analyze the clinical data of 57 patients with PD who underwent bilateral STN-DBS from March 2018 to December 2018. Unified Parkinson’s Disease Rating Scale-Part III (UPDRS-III) score, levodopa equivalent day dose (LEDD), Parkinson’s Disease Quality of Life Scale (PDQ-39) before operation and within 6 months after operation, determine the location of activated contacts and volume of tissue activated (VTA) in the Montreal Neurological Institute (MNI) space, and analyze their correlation with the improvement rate of motor symptoms (UPDRS-III score improvement rate).

**Results:**

After 6 months of follow up, the UPDRS-III scores of 57 patients (Med-off) were improved by 55.4 ± 18.9% (*P*<0.001) compared with that before operation. The improvement rate of PDQ-39 scores [(47.4 ± 23.2)%, (*P* < 0.001)] and the reduction rate of LEDD [(40.1 ± 24.3)%, (*P* < 0.01)] at 6 months postoperation were positively correlated with the improvement rate of motor symptoms (Med-off)(PDQ-39:*r* = 0.461, *P*<0.001; LEDD: *r* = 0.354, *P* = 0.007), the improvement rate of UPDRS-III (Med-off) and the Z-axis coordinate of the active contact in the MNI space were positively correlated (left side: *r* = 0.349,*P* = 0.008;right side: *r* = 0.369,*P* = 0.005). In the MNI space, there was no correlation between the UPDRS-III scores improvement rate (Med-off) at 6 months after operation and bilateral VTA in the STN motor subregion, STN associative subregion and STN limbic subregion of the active electrode contacts of 57 patients (all *P* > 0.05). At 6 months after surgery, the difference between the Z-axis coordinate in the different improvement rate subgroups(<25, 25 to 50%, and>50%) in the MNI space was statistically significant (left side: *P* = 0.030; right side: *P* = 0.024). In the MNI space, there was no statistically significant difference between the groups in the VTA of the electrode active contacts (all *P* > 0.05).

**Conclusion:**

STN-DBS can improve the motor symptoms of PD patients and improve the quality of life. The closer the stimulation is to the STN dorsolateral sensorimotor area, the higher the DBS is to improve the motor symptoms of PD patients.

## Background

PD is a neurodegenerative disease common in middle-aged and elderly people, and deep brain stimulation (DBS) is an accepted treatment at an advanced stage [1, 2]. Studies have shown that subthalamic nucleus (STN) DBS can improve dyskinesias and improve the quality of life (QOL) in PD patients [3–6]. Every now, the STN is publicly preconceived the target of choice [4]. Studies have shown that the improvement in postoperative motor improvement depends in particular on age and disease duration [6] and preoperative response to dopaminergic drugs [7], this makes it critical to screen for the right DBS candidates. In the past, optimizing the parameters of DBS postoperative programming has been proved to be an important factor to improve the therapeutic effect on PD. However, the most important factors that determine the improvement of DBS on motor symptoms are precise stimulation targets and effective stimulation volume, that is, the position of the electrode active contact in STN and VTA [8, 9]. The purpose of this study was to investigate the relationship between the position of bilateral STN-DBS location of active contacts and the clinical efficacy of STN-DBS on motor symptoms in PD patients.

## Methods

Between March and December 2018, a total of 57 subjects were hospitalized at the Department of Neurosurgery of the Beijing Tiantan Hospital affiliated to Capital Medical University, the First Hospital of Hebei Medical University for optimizing a previously performed STN-DBS.

The 57 patients were selected from partial database data, not all patients at the same time. All patients received bilateral stimulation. All patients and their families (spouse or children) have informed consent and signed an informed consent form, the study was approved by the Medical Ethics Committee of Beijing Tiantan Hospital affiliated to Capital Medical University, and the ethics committee approved this form of proxy consent.

### Patient selection

Evaluations were executed by neurologists specialized in movement disorders. Patients with advanced idiopathic PD diagnosed based on the diagnostic criteria for PD in China (2016 edition) and PD surgical treatment evaluation criteria [10]. All patients underwent preoperative testing and analyzed the levodopa challenge test (LCT), confirming that levodopa response needs to be improved by at least 30%, and those who had complete imaging and scoring data and could follow up regularly. Morphologic MRI is performed to exclude patients with severe cerebral atrophy, ischemic disease, and severe cognitive impairment and mental illness.

### Surgical procedures

Surgical procedures were carried out as previously described [11, 12]. An image fusion procedure (3 T MRI and 1.5 T MRI) is commonly used by our group. The Leksell stereotactic frame was placed (Elekta Instruments AB, Stockholm, Sweden) on the day of surgery. A contrast-enhanced full head computerized tomography (CT) scan was carried out and the images were fused with a preoperative frameless MRI including the new planning. All images were imported into Surgiplan (Elekta Instrument AB) and were reformatted. The coordinates of the target and the entrance trajectory were defined by directly visualizing the STN on the image fusion of a CT and preOP MRI, based on MRI T2 DESS (double-echo at steady state). Microelectrode recording (MER) (FHC, Frederic Haer Company, Boston, Massachusetts) and stimulation with neurological response examination were performed. The electrodes were implanted under local anesthesia. The STN target coordinates were 2–3 mm posterior to the mid-intercommissural point (MCP), 11–13 mm lateral to AC-PC and 4–6 mm below the AC-PC [12]. The optimal track (best effects on motor symptoms with the lowest stimulation intensity and largest safety margin) was chosen for each side. Quadripolar electrodes (model L301, PINS Medical, Beijing, China or model 3389 s, Medtronic, Minneapolis, MN, USA), were bilaterally implanted into the STN under local anesthesia in one session. Macro stimulation was used to re-verify target accuracy, symptom improvement and side effects. At the end of the surgical procedure, a implantable pulse generator (IPG) (G102 or G102R (PINS Medical) or Activa RC or Activa PC (Medtronic)) was implanted under general anesthesia on the same day. Postoperative CT scans was performed after surgery on the same day to exclude cerebral hemorrhage and to verify the exact location of the electrodes by merging with the preoperative MR images.

### Stimulation programming

One month after the operation, the IPG was turned on and programmed [13]. DBS was activated with 60 μs,130 Hz and 1.5–2.0 V as standard pulse parameters. The contacts on each electrode were tested and the best stimulation parameters, when the patient achieved satisfactory improvement with minimal side effects were selected. In subsequent follow-up, a regular adjustment of stimulation settings (Voltage) and the levodopa equivalent day dose (LEDD) until optimal control of symptoms was established. Some patients use bipolar or double negative stimulation.

### Clinical evaluation

The study with four assessments, one baseline or screening assessment before surgery, one at 1 month after surgery, one at 3 months after surgery and finally one at 6 months after surgery. Demographic characteristics (age, gender, age at onset, duration of the disease) and disease severity, assessed by the UPDRS-III (3rd edition), ranging from 0 (no impairment) to 108 (maximum impairment),were recorded for all patients. The Hoehn-Yahr scale was used for disease staging. LEDD calculated based on a previously published algorithm combining dopamine agonist daily dose with levodopa daily dose. Quality of life (QOL) was assessed using PDQ-39, ranging from 0 (no impairment) to 124 (maximum impairment). Postoperative motor symptom improvement rate (%) = (preoperative UPDRS-IIIscores-postoperative UPDRS-III scores)/preoperative UPDRS-III scores× 100%. The drug improvement rate was the result of the preoperative levodopa challenge test. The drug improvement rate (%) = (UPDRS-III baseline scores before taking the drug-UPDRS-III lowest scores after taking the drug) / UPDRS-III baseline scores before taking the drug× 100%.Berg Balance Scale score improvement rate (%) = (postoperative BBS score-preoperative BBS score) / preoperative BBS score × 100%. FOG-Q score improvement rate (%) = (preoperative FOG-Q score-postoperative FOG-Q score) / preoperative FOG-Q score × 100%.

### The position of DBS location of active contacts and VTA estimation

(1) DBS electrode location [8, 14]: Use Lead-DBS software (developed by the Department of Movement Disorders, Department of Neuromedicine, Berlin Charity University, Germany) to locate the DBS electrodes. (2) VTA: The volume of STN in the Montreal Neurological Institute (MNI) standard space is defined by the DISTAL atlas, and the VTA is calculated after verifying the electrode position [15, 16].

### Statistical analyses

All statistical analyses were performed using SPSS 25.0 (v25.0.0.0,SPSS Inc., Chicago/Illinois/USA). Continuous variables that followed, or approximately followed, a normal distribution are presented as mean ± standard deviation($\overline{\chi}$ ±s). Continuous variables that did not follow a normal distribution are presented as the median (M) and interquartile range (IQR). The Friedman test was used for continuous variables that did not follow a normal distribution and the Kruskal-Wallis rank sum test was used for comparison between multiple groups. Categorical variables are presented as constituent ratios or percentages, and chi-square tests were used for comparison between groups. Through the Pearson correlation analysize the relationship between the improvement rate of UPDRS-III scores, LEDD change rate, the improvement rate of PDQ-39 scores, the VTA, the coordinates of the electrode activate contacts, the VTA, and the distance from the electrode activate contacts to the STN motor subregion, associative subregion, and limbic subregion were discussed. Pearson correlation analysis was used to determine which factors are associated with improvement of anxiety and depression after DBS. The statistical significance threshold was fixed at *P* < 0.05.

## Results

### Patient population

According to the above criteria, a total of 57 patients were included. Among them, 34 males (59.6%) and 23 females (40.4%); mean age was (64.1 ± 8.0) (46–82); mean onset age (54.0 ± 8.1) (35–73); mean disease duration (10.1 ± 5.1) (2–23). The LEDD of 57 patients before surgery was (866.3 ± 357.0) (125–1625) mg/d; the preoperative Hoehn-Yahr stage was (2.9 ± 0.3) (2–4).

### Clinical outcomes

#### DBS on PD patients with motor symptoms and its correlation analysis results

**Fig. 1:**
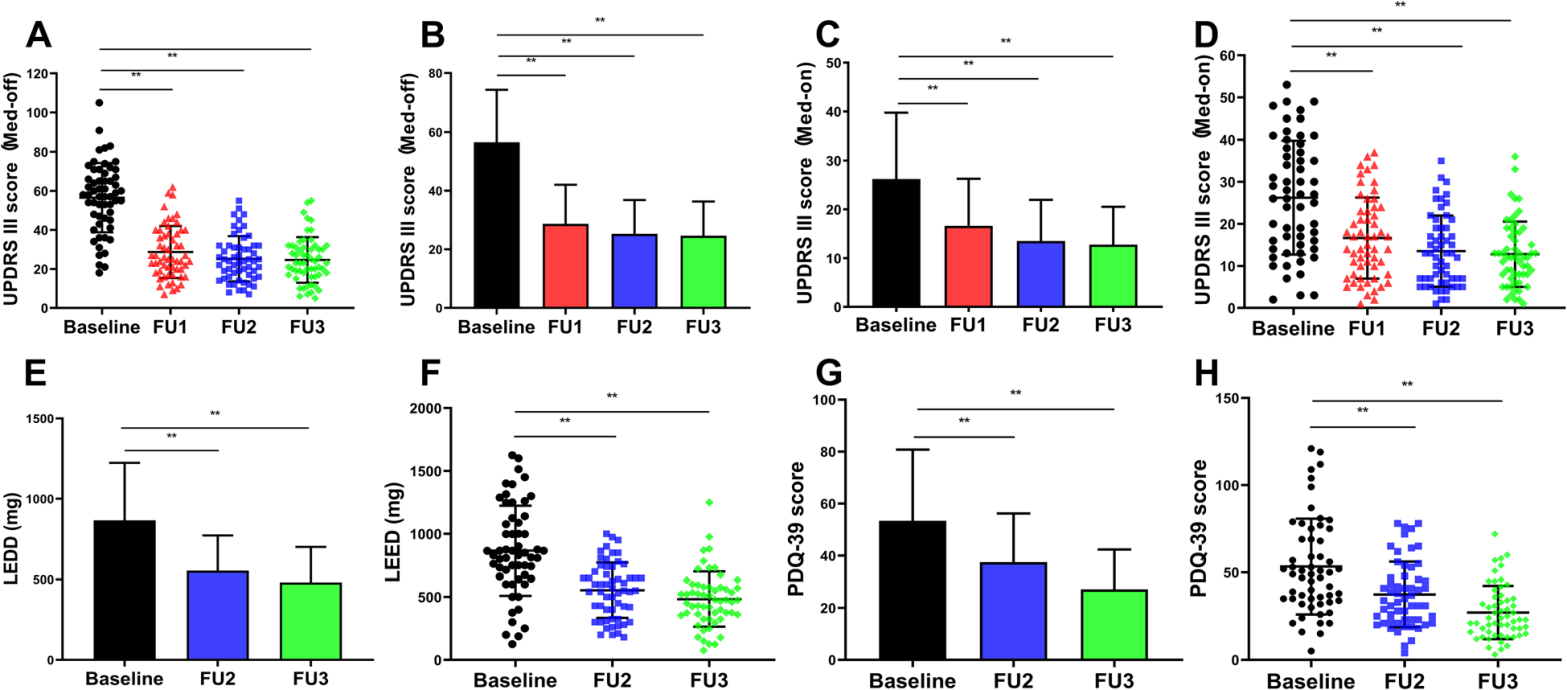
Comparison of preoperative and postoperative HAMA and HAMD scores: **a**, **b** UPDRS-III scores (Med-OFF) were improved by 55.4% follow-up 6 months after surgery; **c**, **d** UPDRS-III scores (Med-OFF) were improved by 44.6% follow-up 6 months after surgery; **e**, **f** LEDD was decreased to 40.1%.(G-H)PDQ-39 scores were improved by 47.4%; (*:*P* < 0.05;**:*P* < 0.001); (STN-DBS: subthalamic nucleus - deep brain stimulation, UPDRS-III: Unified Parkinson’s Disease Rating ScaleIII, BBS: Berg Balance Scale, LEDD: Levodopa equivalent dose, PDQ-39: 39-Item Parkinson’s Disease Questionnaire) [Baseline: baseline; FU1: 1 month after surgery; FU2: 3 months after surgery; FU3: 6 months after surgery]

Comparisons between preoperative and postoperative (1, 3 and 6 months after surgery) clinical stages are summarized in Table 1 and Fig. 1. After 6 months of follow up, the UPDRS-III scores of 57 patients (Med-off) were improved by 55.4 ± 18.9% (*P*<0.001) compared with preoperatively. The improvement rate of PDQ-39 score [(47.4 ± 23.2)%] and the reduction rate of LEDD [(40.1 ± 24.3)%] at 6 months after surgery.

**Table 1:** Comparison of preoperative and postoperative clinical state [M (IQR)]

Time	Medication off		Medication on				
	UPDRS-III (0 ~ 108)	BBS (0 ~ 56)	UPDRS-III (0 ~ 108)	BBS (0 ~ 56)	LEDD (mg)	PDQ-39 (0 ~ 124)	FOG-Q
Preoperative	60.0 (23)	44 (11)	26.2 (23)	52 (7)	831 (453)	49 (38)	14 (12)
Postoperative							
1 month	35.0 (22)	48 (11)	15.0 (16)	53 (5)	–	–	9 (12)
3 month	31.5 (12)	48 (10)	13.0 (13)	53 (5)	550 (357.5)	34 (25)	9 (12)
6 month	31.0 (17)	48 (12)	12.0 (11)	54 (5)	475 (220.5)	23 (20)	9 (10)
Total *P*	<0.001	0.084	<0.001	0.058	<0.001	<0.001	0.068
χ^2^	109.966	35.705	74.042	29.351	62.000	104.246	7.131
*P*_1_	<0.001	–	<0.001	–	–	–	–
*P*_2_	<0.001	–	<0.001	–	< 0.010	< 0.010	–
*P*_3_	<0.001	–	<0.001	–	< 0.010	< 0.010	–

*P*_1_ value is the result of comparison between 1 month and preoperative, *P*_2_ value is the result of comparison between 3 months and preoperative, *P*_3_ value is the result of comparison between 6 months and preoperative; *STN-DBS* Subthalamic nucleus - deep brain stimulation, *UPDRS-III* Unified Parkinson’s Disease Rating ScaleIII, *BBS* Berg Balance Scale, *LEDD* Levodopa equivalent dose, *PDQ-39* 39-Item Parkinson’s Disease Questionnaire, *FOG-Q* Freeze of gait questionnaire; except LEDD unit is mg, all other index units are points

**Fig. 2:**
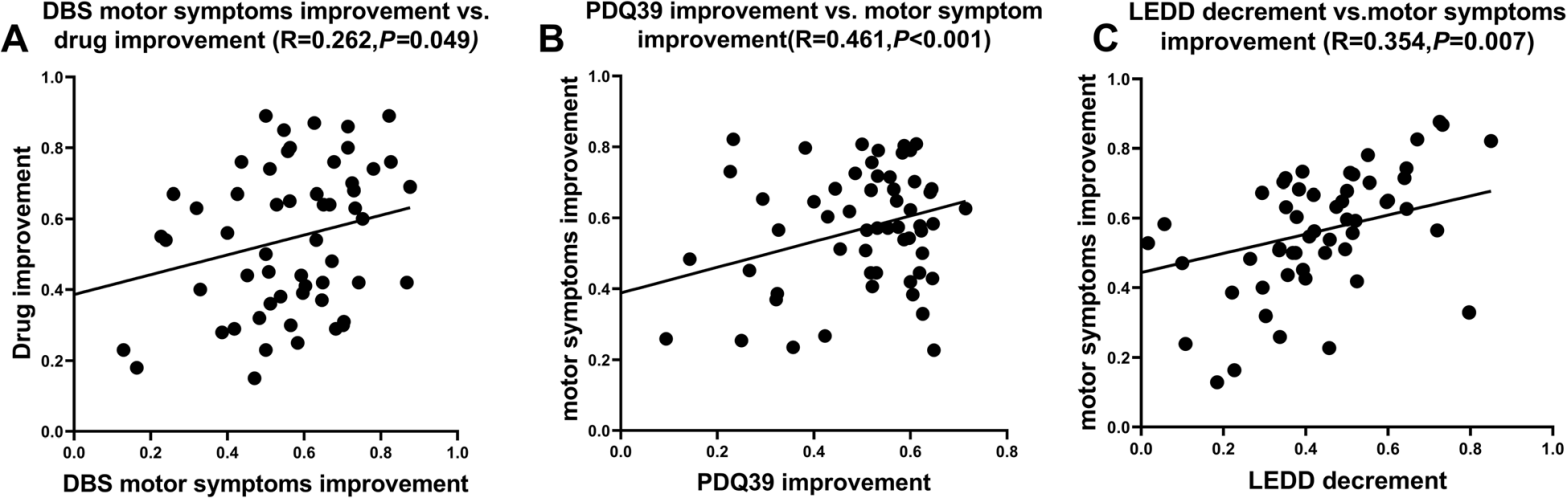
Correlation between the improvement rate of DBS motor symptoms and drug improvement rate (the reduction rate of LEDD), the improvement rate of PDQ39 scores and the decement of LEDD and the improvement rate of motor symptoms: 6 months after surgery, 57 PD patients after STN-DBS. **a** DBS motor symptoms improvement rate (UPDRS-III scores in Med off) was positively correlated with drug improvement rate. **b** The improvement rate of PDQ39 scores was positively correlated with the improvement rate motor symptoms. **c** The decrease of LEDD is positively correlated with the improvement rate of motor symptoms . (STN-DBS: subthalamic nucleus-deep brain stimulation, UPDRS-III: Unified Parkinson’s Disease Rating Scale III, BBS: Berg Balance Scale, LEDD: Levodopa daily equivalent dose, PDQ-39: 39-Item Parkinson’s Disease Questionnaire)

The improvement rate of motor symptoms (UPDRS-III scores) (Med-off) in 57 patients 6 months after operation was positively correlated with the reduction rate of LEDD (*r* = 0.262, *P* = 0.049) (Fig. 2a). At 6 months after surgery, the improvement rate of the PDQ39 scores of 57 patients was positively correlated with the improvement rate of motor symptoms (Med-off) (*r* = 0.461, *P* < 0.001) (Fig. 2b); the reduction rate of LEDD was positively correlated with the improvement rate of motor symptoms (Med-off) (*r* = 0.354, *P* = 0.007) (Fig. 2c).

#### Post-operative DBS setting

Of the 57 patients, 3 (5.3%) required bipolar stimulation, 2 (3.5%) required bipolar negative stimulation, and the remaining 52 patients (91.2%) all received unipolar stimulation. The stimulation parameters of 57 patients: the voltage is (2.04 ± 0.57) V (0.8 ~ 3.0 V), the pulse width is (64 ± 10) μs (50 ~ 90 μs); the frequency is (135 ± 14) Hz (110 ~ 175 Hz).

#### The active contact locations in MNI space and its relationship with the improvement rate of motor symptoms in PD patients

The electrode active contacts: left side: 10 cases with contact 1, 42 cases with contact 2, 5 cases with contact 3; right side: 10 cases with contact 1, 35 cases with contact 2, 11 cases with contact 3 and 1 cases with contact 4(Contact 1 refers to the most ventral contact and contact 4 the most dorsal one).

**Fig. 3:**
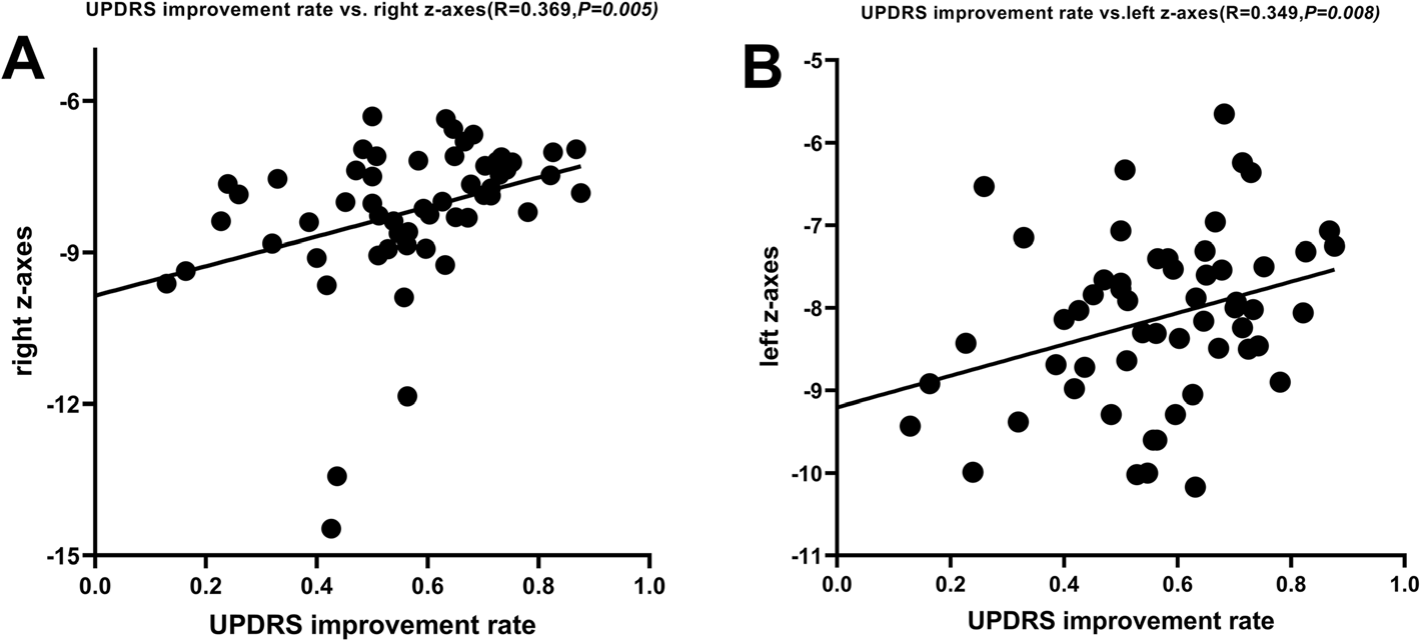
Correlation between the improvement rate of the UPDRS-III scores and the Z-axis coordinate of the active contacts in the MNI space: **a**, **b** The improvement rate of the UPDRS-III scores (Med-off) and the Z-axis of the active contacts in the MNI space are positively correlated (right side: *r* = 0.369, *P* = 0.005; left side: *r* = 0.349, *P* = 0.008) (UPDRS-III:Unified Parkinson’s Disease Rating ScaleIII; MNI:Montreal Neurological Institute)

1. The mean coordinates of active contacts in MNI and AC-PC (anterior commissure- posterior commissure) of 57 patients (Table 2). Six months after surgery, the improvement rate of UPDRS-III (Med-off) and the active contact in MNI the Z-axis of the position is positively correlated (right side: *r* = 0.369,*P* = 0.005;left side: *r* = 0.349,*P* = 0.008) (Figs. 3a.b and 4a.b). This indicates that the higher the z-axis (closer to the dorsal STN), the higher the DBS UPDRS-III improvement rate.
2. In the MNI space, the mean distance from the active contact to the STN motor subregion, STN associative subregion, STN limbic subregion [M (IQR)] was: ①left side: 0.1 (0.5) mm, 0.8 (1.3) mm, 1.0 (1.0) mm.②right side: 0.2 (0.6) mm, 1.1 (1.1) mm, 0.8 (1.4) mm; In the MNI space, there was no correlation between the improvement rate (Med-off) of the UPDRS-III scores in 57 patients 6 months after operation and the electrode active contact to the STN motor subregion (left side: *r* = − 0.152,*P* = 0.259;right side: *r* = − 0.202,*P* = 0.652), STN associative subregion (left side: *r* = − 0.057,*P* = 0.671;right side: *r* = − 0.219, *P* = 0.101) and STN limbic subregion (left side: *r* = 0.100, *P* = 0.461;right side: *r* = 0.241, *P* = 0.071).
3. In the MNI space, the active contact VTA in the STN motor subregion, STN associative subregion, STN limbic subregion were: ①left side: 18.5 (7.5) mm^3^, 13.7 (8.4) mm^3^, 14.7 (8.2) mm^3^;②right side: 16.2 (6.3) mm^3^, 8.3 (9.9) mm^3^, 14.3 (9.0) mm^3^. In the MNI space, there was no correlation between the improvement rate (Med-off) of the UPDRS-III scores in 57 patients 6 months after operation and the VTA in the STN motor subregion (left side: *r* = 0.051,*P* = 0.705;right side: *r* = 0.090,*P* = 0.507),in STN associative subregion (left side: *r* = − 0.113,*P* = 0.403;right side: *r* = 0.205,*P* = 0.127),in STN limbic subregion (left side: *r* = − 0.108, *P* = 0.424;right side: *r* = − 0.236,*P* = 0.077).

**Table 2:** 57 patients cartesian coordinates of active DBS contacts (mm)

Axis	Active contact [mm] in MNI				Active contact [mm] in AC-PC			
	Left		Right		Left		Right	
	Mean	SD	Mean	SD	Mean	SD	Mean	SD
X	−11.5	1.1	12.0	1.1	11.6	1.5	12.0	1.2
Y	−13.5	1.4	−13.5	1.3	−1.7	1.5	−1.6	1.4
Z	−8.2	1.0	−8.3	1.5	−3.9	1.0	−4.0	1.1

*STN-DBS* Subthalamic nucleus deep brain stimulation, *MNI* Montreal Neurological Institute, *AC-PC* Anterior commissure- posterior commissure

## Comparison of DBS active contacts position and VTA in different groups with different improvement rates of motor symptoms

Improvement rates< 25% group:5 cases (8.8%), improvement rates 25–50% group:11 cases (19.3%), improvement rates> 50% group:41 cases (71.9%).

**Fig. 4:**
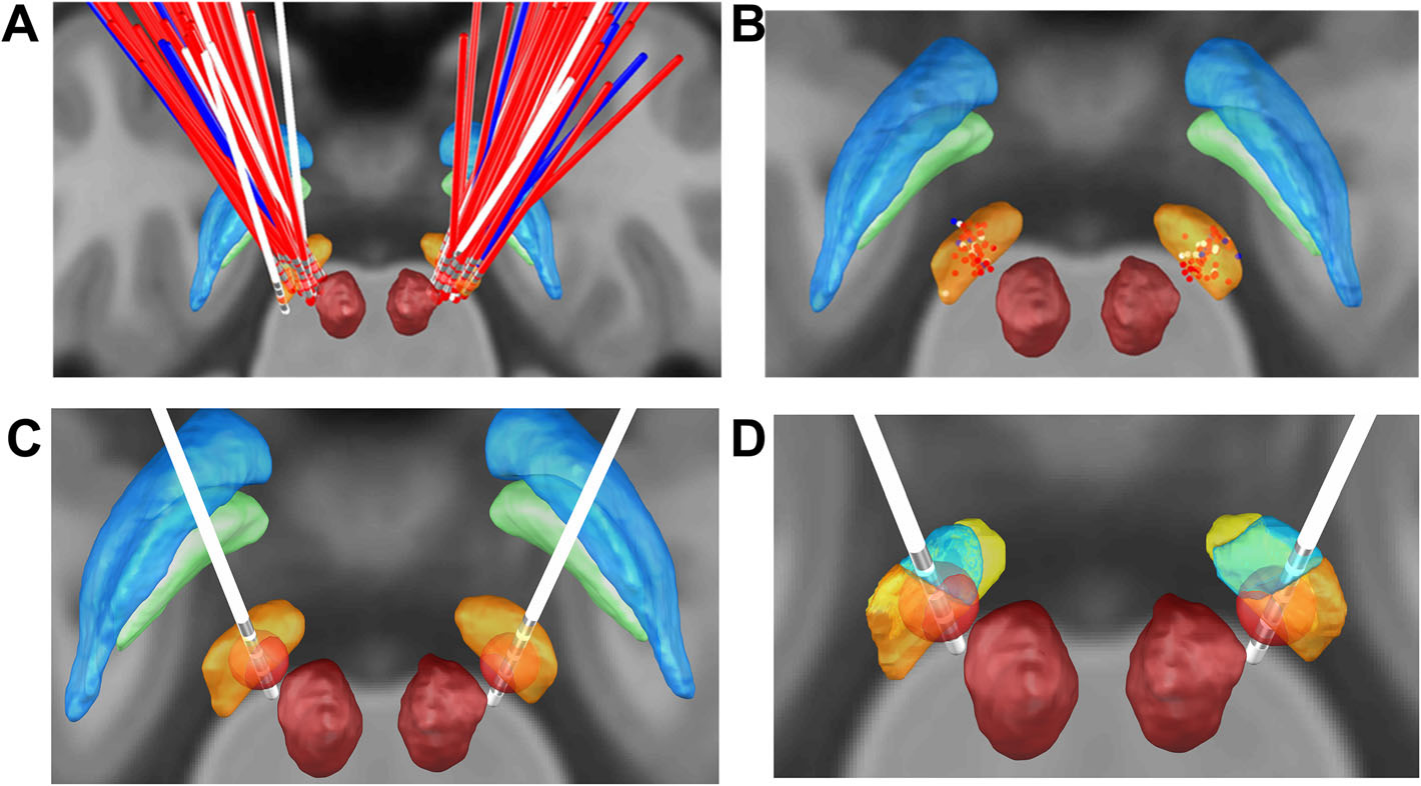
3D illustration of all active electrode contacts: **a**, **b**. Electrode position of 57 PD patients (**a**):Electrode position, posterior view;(**b**):active contact,posterior view; (The blue dots represent group I (DBS improvement rates < 25%), the white dots represent group II (DBS improvement rates between 25 and 50%) and the red dots represent group III (DBS improvement rates> 50%).). **c** relationship between volume of tissue activated (VTA) in STN. (Yellow: STN. Red: red nucleus. Green: Gpi. Blue: Gpe.). **d** relationship between volume of tissue activated (VTA) in STN subregions. (Dark yellow nucleus: STN motor subregion. Blue nucleus: STN associative subregion. Pale yellow nucleus: STN limbic subregion. Red nucleus: red nucleus)

1. The position of the DBS active contacts in groups (Fig. 4a b): In the MNI space, the difference between in the Z-axis coordinate is statistically significant (left side: *P* = 0.030;right side: *P* = 0.024), while on both sides in the X, Y-axis, there was no statistically significant difference between them (all *P* > 0.05, Table 3); In AC-PC space, there was no statistically significant difference between the three groups on the X,Y,Z-axis coordinate (all *P* > 0.05, Table 4), In the MNI space, there was no statistically significant difference between the distances from the active contacts to the STN subregion (all *P* > 0.05, Table 5).
2. The VTA of the electrode active contacts in the MNI space of each group: In the MNI space, there was no statistically significant difference between the groups in the VTA of the electrode active contacts (all *P* > 0.05, Table 6, Fig. 4c.d).

**Table 3:** Comparison results of active contact coordinates in MNI space in different motor symptom improvement rate groups [M (IQR), mm]

Group	Number of cases	Left			Right		
		X	Y	Z	X	Y	Z
Improvement rates < 25% group	5	−12.1 (1.9)	−14.0 (2.6)	−9.4 (1.0)	12.1 (1.9)	−14.7 (2.6)	−9.3 (1.5)
Improvement rates 25–50% group	11	−11.1 (1.9)	− 13.2 (2.6)	−8.1 (1.3)	11.3 (1.9)	− 13.5 (1.9)	−8.4 (2.1)
Improvement rates > 50% group	41	−11.6 (1.2)	− 13.4 (1.7)	−7.9 (1.1)	12.1 (1.6)	− 13.6 (2.2)	−7.8 (1.2)
Kruskal-Wallis χ^2^		2.366	0.461	7.036	1.200	2.261	7.455
*P*		0.306	0.794	**0.030**	0.549	0.323	**0.024**

**Table 4:** Comparison results of active contact coordinates in AC-PC space in different motor symptom improvement rate groups [M (IQR), mm]

Group	Number of cases	Left			Right		
		X	Y	Z	X	Y	Z
Improvement rates < 25% group	5	−10.8 (1.6)	−1.7 (3.2)	−4.5 (0.4)	11.5 (1.6)	−1.9 (2.0)	−4.8 (1.2)
Improvement rates 25–50% group	11	−11.7 (3.2)	− 1.5 (2.5)	− 4.3 (1.9)	11.6 (2.4)	−1.9 (1.2)	− 4.3 (1.5)
Improvement rates > 50% group	41	−11.7 (2.1)	− 1.8 (1.6)	−3.7 (1.6)	12.3 (1.8)	− 1.7 (2.1)	− 3.8 (1.2)
Kruskal-Wallis χ^2^		1.888	0.529	1.970	1.039	0.194	3.847
*P*		0.389	0.768	0.373	0.595	0.908	0.146

**Table 5:** The mean distance of different motor symptom improvement rate groups in the MNI space from the active contacts to the STN subregion [M (IQR), mm]

Group	Number of cases	Left			Right		
		STN motor subregion	STN associative subregion	STN limbic subregion	STN motor subregion	STN associative subregion	STN limbic subregion
Improvement rates < 25% group	5	0.2 (1.2)	1.4 (1.1)	1.5 (1.5)	0.4 (1.2)	1.7 (1.4)	0.4 (1.0)
Improvement rates 25–50% group	11	0.1 (1.4)	0.7 (1.4)	1.9 (0.9)	0.1 (0.6)	0.6 (1.2)	0.5 (0.8)
Improvement rates > 50% group	41	0.1 (0.4)	0.8 (1.2)	0.9 (1.1)	0.2 (0.5)	1.1 (1.1)	0.9 (1.5)
Kruskal-Wallisχ^2^		0.251	1.693	0.255	2.200	4.158	2.908
*P*		0.882	0.429	0.881	0.333	0.128	0.234

**Table 6:** The mean VTA of different motor symptom improvement rate groups in the MNI space [M (IQR), mm^3^]

Group	Number of cases	Left			Right		
		STN motor subregion	STN associative subregion	STN limbic subregion	STN motor subregion	STN associative subregion	STN limbic subregion
Improvement rates < 25% group	5	12.9 (16.6)	9.1 (13.6)	10.6 (9.5)	15.6 (5.3)	5.6 (7.4)	15.7 (8.8)
Improvement rates 25–50% group	11	18.2 (6.3)	15.5 (8.0)	15.6 (10.0)	16.2 (8.0)	12.1 (9.9)	15.2 (5.1)
Improvement rates > 50% group	41	20.1 (13.0)	13.4 (8.3)	14.7 (8.2)	17.1 (10.8)	8.6 (9.9)	12.6 (10.1)
Kruskal-Wallis χ^2^		1.129	1.544	0.140	0.958	3.945	3.613
*P*		0.569	0.462	0.933	0.619	0.139	0.164

## Discussion

STN-DBS has a good effect on PD motor symptoms [17]. In our study, compared to the baseline, STN-DBS improved UPDRS-III scores and major motor function, both Med-on and Med-off postoperatively. These results demonstrate that DBS has a unique advantage in relieving motor symptoms, the patient’s scores in Med-on / Stim-on postoperatively were lower than the scores in Med-on preoperatively; it has a better effect on improving motor symptoms of PD patients. In addition, the most effective contacts were dorsal contacts, this is similar to reports that the contact selection is dorsal to the STN [18]. In our study, we observed a large reduction in LEDD 6 months after operation, LEDD reduction was more than 40% compared to the preoperative dose, which was related to the improvement rate of motor symptoms in DBS (UPDRS-III scores reduced by 55.4%), Which was consistent with a 19 to 80.7% reduction in drug dose and 53–92% improvement in dyskinesia scores [19, 20]. The LEDD reduction rate was positively correlated with the improvement rate of motor symptoms (*r* = 0.354, *P* = 0.007). We think that the better the effect of DBS on improving patients’ motor symptoms, the more LEDD is reduced. Through the PDQ-39 assessment, the QOL (quality of Life) of our patients improved by 47.4% overall, which also proved the good effect of STN-DBS. This result is consistent with previous studies, with an improvement in quality of life from 30.2 to 50.6% [21]. In our study the PDQ39 improvement rate was positively correlated with the improvement rate of motor symptoms, (*r* = 0.461,*P* < 0.001),the better the effect of DBS on improving motor symptoms, the better the QOL of patients.

### Active contact location

We observed that electrode active contacts in STN-DBS patients were mainly distributed in dorsolateral STN. As we all know, the dorsolateral STN is involved in motor function, and the dorsolateral STN serves as the target region for STN-DBS in PD patients [22]. Stimulation of the dorsolateral STN (sensory motor function area) is expected to disrupt pathological neuronal motor activity or afferent fibers and improve clinical symptoms. In our study, the improvement rate of UPDRS-III scores of more than 50% was basically concentrated in the dorsolateral part of STN, the active contacts location of patients with the improvement rate of UPDRS-III scores 25 to 50% was more concentrated in the middle part of STN, the active contacts position of patients with the improvement rate of UPDRS-III scores less than 25% was more concentrated in the ventral part of STN. After statistical analysis, it was found that only the z-axis coordinate in MNI was significantly different. Optimal location of DBS stimulation within STN: the dorsolateral part of STN is traditionally considered to represent the optimal location of the motor region and stimulation [23]. So far, this part can only be confirmed by intraoperative electrophysiology, which shows an increase in βoscillation activity [24]. The results of our study support the conclusion that the position of the electrode active contacts help to judge the motor effect of STN-DBS. This study found that the improvement rate of motor symptoms is related to the reduction of LEDD, but the correlation coefficient is only 0.262, which needs further research.

### .VTA

The effect of programmed parameters (voltage, pulse width, frequency) on the efficacy of DBS surgery is critical. The therapeutic effect is not only on the single contact, but also on the larger electric field range than the contact. Therefore, Andreas Horn’s method of VAT calculation [15] was used to analyze the stimulation parameters of postoperative active contacts and calculate the correlation between the VTA and the UPDRS motor scores. We used the VAT calculation to evaluate the clinical efficacy of STN-DBS in PD patients, although the difference between the VTA of the electrode activate contacts in the MNI space of each group was not statistically significant (all *P* > 0.05), however, it was found that the higher the VTA of the electrode activate contact of the patient in the STN motor subregion, the higher the improvement of motor symptoms. The difference is not statistically significant and may be related to the sample size of this study. It may be due to the individual differences in the anatomical structure of the target area, due to the difference in the myelin sheath and diameter of the axons in the brain tissue, the response to stimulation may be different, the conductivity and dielectric value of the tissue are different, and the transmission of excitability and excitement may be affected. Influences. In addition, impedance, tissue capacitance and other factors will cause the difference of results.

## Limitations

(1) It is a retrospective analysis of a small sample of 57 patients, the sample is small; (2) The average follow-up period is half a year and the time is short; (3) The treatment mechanism of DBS in this study has not been clarified. Despite these limitations, our results further confirm that DBS electrode active contacts located dorsolateral to STN can achieve better clinical efficacy and are proportional to the percentage of VTA located in STN motor subregion. Therefore, direct functional evidence supports only a mild dorsal- ventral gradient of STN DBS motor effects, and does not support strict dorsal- ventral dissociation.

## Conclusion

STN-DBS can improve the motor symptoms of PD patients and improve the quality of life. The closer the stimulation is to the STN dorsolateral sensorimotor area, the higher the DBS is to improve the motor symptoms of PD patients.

## Data Availability

The datasets used and/or analyzed during the current study not publicly available due to privacy reasons of patients, but are available from the corresponding author on reasonable request.

## Abbreviations

PD: Parkinson’s disease
STN: Subthalamic nucleus
DBS: Deep brain stimulation
UPDRS-III: Unified Parkinson’s Disease Rating Scale-Part III
LEDD: Levodopa equivalent day dose
QOL: Quality of life
PDQ-39: 39-Item Parkinson’s Disease Questionnaire
VTA: Volume of tissue activated
MNI: Montreal Neurological Institute
LCT: Levodopa challenge test
CT: Computerized tomography
MER: Microelectrode recording
Med-off: Medication-off
Med-on: Medication-on
MCP: Mid-intercommissural point
AC-PC: Anterior Commissure- Posterior Commissure
IPG: Implantable pulse generator
